# Antiseptics and mupirocin resistance in clinical, environmental, and colonizing coagulase negative *Staphylococcus* isolates

**DOI:** 10.1186/s13756-023-01310-3

**Published:** 2023-10-04

**Authors:** Enas Mamdouh Hefzy, Tharwat E. E. Radwan, Basma M. M. Hozayen, Eman E. Mahmoud, Mahmoud A. F. Khalil

**Affiliations:** 1 Medical Microbiology and Immunology Department, Faculty of Medicine, Fayoum University, Fayoum, 63514 Egypt; 2 Botany Department, Faculty of Science, Fayoum University, Fayoum, Egypt; 3 Department of Clinical and Chemical Pathology, Faculty of Medicine, Fayoum University, Fayoum, Egypt; 4 Microbiology and Immunology Department, Faculty of Pharmacy, Fayoum University, Fayoum, Egypt

**Keywords:** Antiseptic resistance, CoNS, Mupirocin resistance, *S. chromogene*, *S. epidermidis*

## Abstract

**Background:**

Coagulase-Negative *Staphylococci* (CoNS) are opportunistic and nosocomial pathogens. The excessive use of antimicrobial agents, including antiseptics, represents one of the world’s major public health problems. This study aimed to test the susceptibility of CoNS to antiseptics.

**Methods:**

Out of 250 specimens collected from different sections of the hospital, 55 samples were identified as CoNS, categorized into three groups based on their sources: environmental samples (n = 32), healthcare worker carriers samples (n = 14), and clinical infection samples (n = 9). Isolates were examined for susceptibility to antibiotics and antiseptics, such as benzalkonium chloride (BC), cetyltrimethylammonium bromide (CTAB), and chlorhexidine digluconate (CHDG). Mupirocin and antiseptic resistance genes, as well as the *mecA* gene, were detected using polymerase chain reaction. CoNS isolates with notable resistance to antiseptics and antibiotics were identified using the API-Staph system.

**Results:**

A high frequency of multidrug resistance among CoNS clinical infection isolates was observed. Approximately half of the CoNS isolates from healthcare workers were susceptible to CHDG, but 93% were resistant to BC and CTAB. The frequency of antiseptics and antibiotics resistance genes in CoNS isolates was as follows: *qacA/B* (51/55; 92.7%), *smr* (22/55; 40.0%), *qacG* (1/55; 1.8%), *qacH* (6/55; 10.9%), *qacJ* (4/55; 7.3%), *mecA* (35/55; 63.6%), *mupB* (10/55; 18.2%), and *mupA* (7/55; 12.7%). A significant difference in the prevalence of *smr* gene and *qacJ* genes between CoNS isolates from healthcare workers and other isolates was reported (P value = 0.032 and ˂0.001, respectively). Four different CoNS species; *S. epidermidis, S. chromogene, S. haemolyticus*, and *S. hominis*, were identified by API.

**Conclusions:**

CoNS isolates colonizing healthcare workers showed a high prevalence of antiseptic resistance genes, while clinical infection samples were more resistant to antibiotics. CHDG demonstrated greater efficacy than BC and CTAB in our hospital.

**Supplementary Information:**

The online version contains supplementary material available at 10.1186/s13756-023-01310-3.

## Background

Coagulase-negative *Staphylococci* (CoNS) are among the microbiota found on mucous membranes and skin, causing internal infections in both animals and humans. Insufficient hand washing, contaminated surfaces, and poor tool sterilization are key factors contributing to the spread of CoNS, which can be transmitted through the hands of healthcare workers [1]. CoNS-associated infections are linked to the use of implanted or indwelling medical tools, resulting in significant medical and economic losses [2].

Antiseptics consist of various chemical compounds, such as quaternary ammonium compounds (QACs) and biguanides. They are used in medical staff hand washes, preoperative skin preparation solutions, and in various industries as detergents, soap, and mouthwash. Antiseptics are also employed to disinfect mucosa and skin during invasive procedures and surgery. However, excessive use of antiseptic agents without following instructions, whether in hospitals or the community, contributes to the development of resistance to antiseptics [3].

Chlorhexidine digluconate (CHDG) is a cationic biguanide compound used for skin antisepsis, wound dressings, and catheters to prevent healthcare-associated infections [4, 5]. Benzalkonium chloride (BC) and cetyltrimethylammonium bromide (CTAB) are particularly safe antibacterial cationic surfactants, and they are cost-effective in maintaining increased industrial productivity [6, 7]. Cationic surfactants with antimicrobial activity, having a hydrophobic alkyl chain and a hydrophilic quaternary ammonium group, are commonly used for cleanliness and disinfection in various settings, including hospitals and the food industry. It has been hypothesized that their widespread and increasing use has contributed to the emergence of antimicrobial cationic surfactant-resistant bacteria, which, under certain circumstances, may become multidrug-resistant [8].

Studies have shown that the presence of plasmid-mediated antiseptic resistance genes, such as *qacA/B, smr, qacG, qacH*, and *qacJ*, increases resistance to antiseptics. The co-existence of antiseptic resistance genes and antibiotic resistance genes on plasmids further enhances resistance in pathogens [9]. Bacteria can resist antiseptic attacks through intrinsic (natural) mechanisms or acquired resistance via mutation or plasmid acquisition [10]. A variety of mechanisms, such as biofilm formation, the efflux pump system, enzymatic deactivation, alterations in membrane permeability, and target site modification [10, 11], contribute to bacterial resistance to antiseptics. CoNS have been identified as a reservoir of resistance genes that can transfer to other pathogens, such as *Staphylococcus aureus (S. aureus*) [1].

For the prevention of nosocomial infections, it becomes essential to study epidemiological data on antiseptic susceptibility and the spread of antiseptic resistance genes [9]. This study aimed to test the susceptibility of CoNS isolated from various niches, including the hospital environment, clinical infections, and colonized healthcare workers, to different antiseptics frequently used in Egyptian hospitals.

## Methods

Hospitals frequently utilize antiseptics as biocides to prevent the spread of infection. We aimed to examine the susceptibility of Coagulase-negative *Staphylococci* (CoNS) to different antiseptics commonly used in Egyptian hospitals. CoNS isolates from the environment, colonized healthcare workers, and clinical infection samples were collected, identified, and tested.

### Study settings

This descriptive cross-sectional study was designed and conducted under the supervision of the Infection Control Unit at Fayoum University Hospitals between November 2021 and October 2022.

### CoNS isolation and identification

Two hundred and fifty specimens were collected from different departments of the hospital, including environmental samples (n = 183) from high-touch areas, in operating rooms and intensive care units, as defined by the World Health Organization (WHO) [12], healthcare worker samples (n = 52) from the noses and hands of doctors, nurses, and housekeepers, and clinical infection samples (n = 15) from blood cultures or abscesses. Cotton swabs in 2 ml saline solution were used to screen the environment and healthy human carriers. The samples were kept on ice and transported to the laboratory of the medical microbiology department at Fayoum Faculty of Medicine within 2 h. The samples were then processed, cultured onto a mannitol salt agar (MSA) plate (Himedia, India), and incubated for 24 h at 37 °C. Clinical infection samples were collected at the microbiology lab of Fayoum University Hospital, where they were identified as CoNS. CoNS were identified using conventional bacteriological methods [13] and confirmed by molecular methods. Only confirmed CoNS isolates were selected for further characterization and antiseptic susceptibility testing.

### Antibiotic susceptibility testing of identified CoNS isolates and detection of methicillin resistant CoNS (MR-CoNS) isolates

Isolates identified as CoNS were tested for their antibiotic susceptibility using the Kirby–Bauer disk diffusion method. The principles of the Clinical and Laboratory Standards Institute (CLSI) were used, and the results were interpreted accordingly [14, 15]. Identification of methicillin resistance among CoNS (MR-CoNS) isolates was done by using cefoxitin disc diffusion test as recommended by CLSI [15]. *S. aureus* ATCC 25923 was used as a quality control test organism for antibiotic susceptibility testing.

### Antiseptics susceptibility testing of identified CoNS isolates

The broth microdilution method in a 96-well microtiter plate format was used for antiseptics susceptibility testing. The protocols followed the guidelines of the European Committee for Antimicrobial Susceptibility Testing (EUCAST), CLSI, and Wiegand et al. [15–18]. Three types of antiseptics (Sigma Aldrich, St. Louis, USA) were tested against different CoNS isolates: (1) cetyltrimethylammonium bromide (CTAB), CAS (Chemical Abstracts Service) Number: 57-09-0, Potency ≥ 96.0%, powder format, dissolved in water; (2) benzalkonium chloride (BC), CAS Number: 63449-41-2. Potency ≥ 95.0%, semisolid format, dissolved in water); and (3) chlorhexidine digluconate (CHDG), CAS Number: 18472-51-0, Concentration 20% in Liquid format. For broth micro-dilution tests, a stock solution was prepared with a concentration 10 times greater than the greatest concentration to be tested. The fresh antiseptics stock solution was prepared directly before each use [19].

The sterile microtiter plate was labeled with ten respective antiseptic concentrations ranging from 128 mg/L to 0.25 mg/L for all antiseptics and tested against an equal concentration of bacteria (0.5 McFarland Standard) in an equal volume of Muller Hinton broth (Himedia, India). Plates were incubated for 24 h at 37 °C. When no growth occurred in all of the concentrations tested, the minimum inhibitory concentration (MIC) was recorded as less than or equal to the lowest concentration. CHDG resistance was defined as a MIC ≥ 4 µg/ml [20], while for CTAB and BAC, MIC ≥ 0.5 µg/ml [21] and ≥ 3 µg/ml [6], respectively, were considered resistant.

### PCR assays for CoNS identification and detection of antibiotic and antiseptic resistance genes

#### DNA extraction

DNA extraction was performed using the Thermo Scientific GeneJET Genomic DNA Purification Kit (#K0721) (Thermo Fisher Scientific Baltics UAB Company, Vilnius, Lithuania), following the Gram-Positive Bacteria Genomic DNA Purification Protocol.

#### PCR reaction for CoNS identification

CoNS were identified by targeting the *Staphylococcus* 16S rRNA gene as a specific marker for the *Staphylococci* genus and the *nuc* gene that differentiates between *S. aureus* and CoNS. Sterile distilled water was used as a negative control, while DNA extracted from *S. aureus* ATCC 43300 was used as a positive control for *mecA*. The DNA extracted from previously isolated and characterized strains was used as a positive control for antiseptic resistance genes. Amplification parameters were adopted from Mcclure and colleagues with modifications, including an initial denaturation of 5 min at 95 °C, followed by 10 cycles of 95 °C for 30 s, 58 °C for 40 s, and 72 °C for 50 s, and 25 cycles of 95 °C for 30 s, 50 °C for 40 s, and 72 °C for 50 s, with a final extension at 72 °C for 7 min [9].

#### Detection of methicillin resistance, mupirocin resistance, and antiseptic resistance genes by PCR

A multiplex PCR assay targeted the *mecA* gene (a determinant of methicillin resistance), *mupA* and *mupB* genes (mupirocin resistance genes), and *qacA, qacB*, and *smr* (antiseptic resistance genes). Amplification parameters of Mcclure and colleagues with modifications were followed, including an initial denaturation for 5 min at 95 °C, 10 cycles of 95 °C for 30 s, 58 °C for 40 s, and 72 °C for 50 s, and 25 cycles of 95 °C for 30 s, 40 °C for 30 s, and 72 °C for 50 s, with a final extension at 72 °C for 7 min [9].

Each of the antiseptic resistance genes (*qacG, qacH*, and *qacJ*) was detected by a separate PCR. The cycling conditions for *qacG, qacH*, and *qacJ* genes were as follows: an initial denaturation at 94 °C for 10 min, 25 cycles of 95 °C for 60 s, 48 °C for 45 s, 72 °C for 60 s, and a final extension step at 72 °C for 10 min [14].

Primer sequences and expected product sizes for all PCR reactions are shown in Additional Table 1. The PCR amplicons were separated by electrophoresis on a 1.4% agarose gel stained with 0.85 µg/ml ethidium bromide, visualized using a Clear View UV Transilluminator (Cleaver Scientific Ltd, United Kingdom). All oligonucleotide primers were synthesized at Thermo Fisher Scientific Company (United Kingdom). The size of the amplicons was estimated by comparison with a size ladder (100 bp DNA Ladder Marker, enzynomics, Korea).

#### API-Staph system for CoNS identification

The multi-drug-resistant CoNS isolates that exhibited high resistance to antiseptics were identified to the species level using the API-Staph system (API-Staph (2019), biomérieux, Paris, France) following the manufacturer’s instructions.

#### Statistical analysis of results by SPSS software

The collected data were processed and analyzed using SPSS 16 (SPSS Inc. Chicago, IL, USA). Categorical data were presented using frequency and percentages. The Chi-square test was used to determine the relationship between quantitative variables. Fisher-Exact test was used for two-by-two tables with expected cell frequency in any cell less than five. A P-value < 0.05 was considered significant.

## Results

### CoNS isolation and identification

Out of the 250 specimens collected, 55 CoNS isolates were identified. Among these, 32 were retrieved from environmental samples, 14 from colonized healthcare workers’ samples, and nine were clinical infection samples (Additional Table 2 provides information about clinical isolates).

### Antibiotic susceptibility testing of CoNS isolates

All CoNS isolates were resistant to penicillin G, with 36 (65.5%) being resistant to cefoxitin, 52 (94.5%) resistant to ampicillin, and 40 (72.7%) resistant to amoxicillin/clavulanic acid. None of the isolates showed resistance to linezolid. The results of antibiotic susceptibility testing of CoNS isolates are shown in Additional Table (3).

This study observed higher resistance to antibiotics in clinical infection samples compared to healthcare worker and environmental samples for all antibiotics, although this difference was significant for clindamycin and rifampicin only (P value = 0.003 & 0.02, respectively). Table (1) describes antibiotic resistance according to the source of isolates.

**Table 1:** Antibiotic resistance according to source of isolates

Antibiotics		Sample Source (N=55)			P value
		Enviromental N = 32	Healthcare Workers N = 14	Clinical infections N = 9	
**Penicillin G (P) (10 µg)**	N(%)	32(100)	14(100)	9(100)	
**Cefoxitin (CX)** (30 µg)	N(%)	23(71.9)	13(92.9)	8(88.9)	0.201
**Oxacillin (OX)** (1 µg)	N(%)	11(34.4)	6(42.9)	6(66.7)	0.221
**Erythromycin (E) **(15 µg)	N(%)	25(78.1)	10(71.4)	9(100.0)	0.227
**Clindamycin (CD) (2 µg)**	N(%)	6(18.8)	4(28.6)	7(77.8)	0.003*
**Trimethoprim/ sulfamethoxazole (COT) (25 µg)**	N(%)	4(12.5)	5(35.7)	4(44.4)	0.064
**Ciprofloxacin (CIP) (5 µg)**	N(%)	3(9.4)	3(21.4)	4(44.4)	0.051
**Tetracycline (TE) (30 µg)**	N(%)	5(15.6)	3(21.4)	1(11.1)	0.79
**Rifampicin (RIF) (5 µg)**	N(%)	3(9.4)	1(7.1)	4(44.4)	0.02*
**Gentamicin (GEN) (10 µg)**	N(%)	2(6.3)	1(7.1)	1(11.1)	0.88
**Cloramaphenicol (C) (30 µg)**	N(%)	2(6.3)	0	0	0.5
**Linezolid (LZ) (30 µg)**	N(%)	0	0	0	
**Teicoplanin (TEI) (30 µg)**	N(%)	3(9.4)	0	0	0.32
**Imipenem (IPM) (10 µg)**	N(%)	2(6.3%)	1(7.1%)	1(11.1%)	
**Amoxicillin.clavulanic acid (AMC) (20/10 µg)**	N(%)	21(65.6)	12(85.7)	8(88.9)	0.2
**cefotaxime (CTX) (30 µg)**	N(%)	20(62.5)	9(64.3)	8(88.9)	0.32

*Significant

### Antiseptics susceptibility testing of CoNS isolates

The minimum inhibitory concentration (MIC) of antiseptics against CoNS isolates was detected according to the breakpoints mentioned. Most CoNS isolates (52/55, 94.5%) were resistant to BC and CTAB, while 44/55 (80%) were resistant to CHDG. The results are shown in Additional Table (4).

We found that the highest resistance to antiseptics was observed among environmental samples. A significant difference in resistance to CHDG was noted between environmental samples and healthcare worker and clinical infection samples (P value = 0.001*) (Table 2).

**Table 2:** Antiseptic susceptibility according to source of isolates

Antiseptics		Sample Source (N=55)					
		Environmental samples (N = 32)		Healthcare workers (N = 14)		Clinical Infections (N = 9)	P Value
**Benzalkonium chloride (BC)**	Sensitive **N (%)**	1(11.1)		1(7)		1(11.1)	0.6
	Resistant **N (%)**	31(96.9)		13(93)		8(11.1)	
**Cetyltrimethy lammonium bromide (CTAB)**	Sensitive **N (%)**	0		1(7)		1(11.1)	0.2
	Resistant **N (%)**	32(100)		13(93)		8(88.9)	
**Chlorhexidine digluconate (CHDG)**	Sensitive **N (%)**	1(3.1)		7(50)		3(33.3)	0.001*
	Resistant **N (%)**	31(96.9)		7(50)		6(66.7)	

*Significant; CHDG resistance is defined as a MIC ≥ 4 µg/ml [18]; CTAB resistance is defined as a MIC ≥ 0.5 µg/ml [19], and BAC with a MIC > 3 µg/ml [6] was considered resistant

### Detection of antibiotics and antiseptics resistance genes by polymerase chain reaction (PCR)

Antibiotics and antiseptics resistance genes *mecA* (112 bp), *mupB* (674 bp), *mupA* (456 bp), *qacA/B* (361 bp), and *smr* (195 bp) were detected by multiplex PCR, while each of *qacG* (275 bp), *qacH* (295 bp), *qacJ* (301 bp) was detected in a separate PCR reaction.

**Fig. 1:**
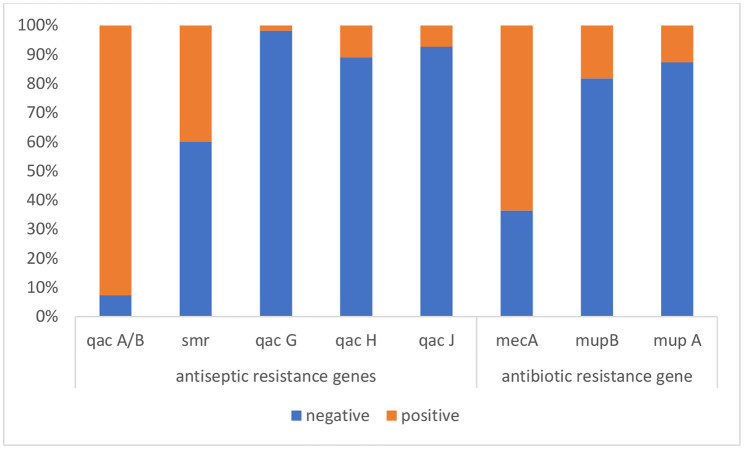
Frequency of antibiotics and antiseptics resistance genes among CoNS isolates

Out of 55 CoNS isolates, 51 (92.7%) had *qacA/B*, 22 (40.0%) had *smr*, one (1.8%) had *qacG*, 6 (10.9%) had *qacH*, and 4 (7.3%) had *qacJ. mecA, mupB*, and *mupA* were detected in 35 (63.6%), 10 (18.2%), and 7 (12.7%) CoNS isolates, respectively. The data is shown in Fig. (1).

By studying the difference in prevalence of antibiotics and antiseptics resistance genes among CoNS isolates according to their source, we found a significantly higher prevalence of *smr, qacJ*, and *mupB* genes among CoNS isolates from healthcare carriers compared to other isolates (P value˂0.001, 0.032, and 0.025, respectively). The data is shown in Table 3.

**Table 3:** Prevalence of antibiotics and antiseptics resistance genes among CoNS isolates according to its source

Antiseptic and Antibiotic Resistance Genes			Sample Source (N = 55)			P value	
			Environmental Samples (N = 32)	Healthcare worker (N = 14)	Clinical Infections (N = 9)		
***qac A/B***	**Negative**	N (%)	3(9.4)	1(7.1)	0	0.633	
	**Positive**	N (%)	29(90.6)	13(92.9)	9(100.0)		
***Smr***	**Negative**	N (%)	25(78.1)	2(14.3)	6(66.7)	< 0.001*	
	**Positive**	N (%)	7(21.9)	12(85.7)	3(33.3)		
***qac G***	**Negative**	N (%)	32(100.0)	13(92.9)	9(92.9)	0.225	
	**Positive**	N (%)	0	1(7.1)	0		
***qac H***	**Negative**	N(%)	29(90.6)	11(78.6)	9(100.0)	0.250	
	**Positive**	N (%)	3(9.4)	3(21.4)	0		
***qac J***	**Negative**	N (%)	32(100.0)	11(78.6)	8(88.9)	0.032*	
	**Positive**	N (%)	0	3(21.4)	1(11.1)		
***mecA***	**Negative**	N (%)	15(46.9)	4(28.6)	1(11.1)	0.112	
	**Positive**	N (%)	17(53.1)	10(71.4)	8(88.9)		
*mup B*	**Negative**	N (%)	30(93.8)	9(64.3)	6(66.7)	0.025*	
	**Positive**	N (%)	2(6.3)	5(35.7)	3(33.3)		
*mup A*	**Negative**	N (%)	27(84.4)	12(85.7)	9(100)	0.45	
	**Positive**	N (%)	5(15.6)	2(14.3)	0(0.0)		

*Significant

### API-Staph system

CoNS isolates that exhibited high resistance to antiseptics and antibiotics were selected for species identification using the API-Staph system. Among the 25 CoNS isolates tested by the API-Staph system, 19 were *S. epidermidis*, four were *S. chromogenes*, one was *S. haemolyticus*, and one was *S. hominis.*

By studying the difference in antibiotic susceptibility between *S. epidermidis* (N = 19) and non-epidermidis CoNS (N = 6), no difference was noted for all antibiotics except for clindamycin (P value = 0.003) (Additional Table 5).

All the CoNS isolates selected for identification by the API-Staph system were resistant to BC and CTAB. By studying the difference in antiseptic susceptibility between *S. epidermidis* and non-epidermidis CoNS, no difference was noted for all antiseptics (P value > 0.05) (Table 4).

**Table 4:** Difference in antiseptic susceptibility between *S. epidermides* and non-epidermides CoNS

Antiseptics		*S. epidermidis* (N = 19)	Non-epidermidis CoNS. (N = 6)	P value
**Benzalkonium chloride (BC)**	Sensitive N (**%)**	0	0	
	Resistant N (**%)**	19 (100)	6 (100)	
**Cetyltrimethyla mmonium** **bromide (CTAB)**	Sensitive N (**%)**	0	0	
	Resistant N (**%)**	19 (100)	6 (100)	
**Chlorhexidine digluconate (CHDG)**	Sensitive N (**%)**	5 (26.3)	1 (16.7)	
	Resistant N (**%)**	14 (73.7)	5 (83.3)	0.9

CHDG resistance is defined as a MIC ≥ 4 µg/ml [18]; CTAB resistance is defined as a MIC ≥ 0.5 µg/ml [19], and BAC with a MIC > 3 µg/ml [6] was considered resistant

No significant difference was noted regarding the presence of antiseptic resistance genes between *S. epidermidis* and non-epidermidis CoNS. The prevalence of antiseptic and antibiotic resistance genes in *S. epidermidis* and non-epidermidis CoNS isolates is reported in Table (5).

**Table 5:** Prevalence of antiseptic and antibiotic resistance genes between *S. epidermides* and non-epidermides CoNS

Antiseptic and Antibiotic Resistance Genes			*S. epidermidis* (N = 19)	Non-epidermidis CoNS. (N = 6)	P value
***qacA/B***	**Positive**	N (%)	19(100)	6(100)	
	**Negative**	N (%)	0	0	
***smr***	**Positive**	N (%)	13(68.4)	2(33.3)	> 0.999
	**Negative**	N (%)	6(31.6)	4(66.7)	
***qac G***	**Positive**	N (%)	18(94.7)	6(100.0)	> 0.999
	**Negative**	N (%)	1(5.3)	0	
***qac H***	**Positive**	N (%)	17(89.5)	5(83.3)	> 0.999
	**Negative**	N (%)	2(10.2)	1(16.7)	
***qac J***	**Positive**	N (%)	16(84.2)	5(83.3)	> 0.999
	**Negative**	N (%)	3(15.8)	1(16.7)	
***mecA***	**Positive**	N (%)	19(100)	2(33.3%)	< 0.001*
	**Negative**	N (%)	0(0.0)	4(66.6%)	
***mupB***	**Positive**	N (%)	18(94.7)	4(66.6%)	< 0.07
	**Negative**	N (%)	1(5.3)	2(33.3%)	
***mup A***	**Positive**	N (%)	19(100)	5(83.3)	< 0.04*
	**Negative**	N (%)	0(0.0)	1(16.6%)	

*Significant

In the current study, 52/55 (94.5%) CoNS isolates harbored at least one antiseptic resistance gene. Additionally, 35 (63.8%) of isolates were identified as methicillin-resistant CoNS (MR-CoNS) by molecular detection of the *mecA* gene. Among the MR-CoNS, 19 were environmental samples (19/32 = 59.4%), 7 were clinical samples (7/9 = 77.8%), and 9 were healthcare samples (9/14 = 64.3%) (Data is not shown in tables). Table (6) displays the frequency of antiseptic resistance genes detected among methicillin-sensitive CoNS (MS-CoNS)(N = 20) and MR-CoNS (N = 35) isolates. Three (3/55) CoNS isolates were resistant to antiseptics but had no antiseptic resistance genes.

**Table 6:** Frequency of antiseptic resistance gene among CoNS isolates

Prevalence of antiseptic resistance genes	Antiseptic resistance genes	Methicillin-sensitive- CoNS (N = 20)	Methicillin-resistant-CoNS (N = 35)
**One gene (29/55)**	(28) *qac A/B*	10	18
	*(1) qac H*	0	1
**Two gene (17/55)**	(15) *qac A/B*, *smr*	4	11
	(2) *qac A/B*, *qac H*	2	0
**Three genes (4/55)**	(1) *qac A/B*, *smr*, *qac G*	0	1
	(1) *qac A/B*, *smr*, *qac H*	0	1
	(2) *qac A/B*, *smr*, *qac J*	0	2
**Four genes (2/55)**	(2) *qac A/B*, *smr*, *qac H, qac J*	1	1
**Zero gene (3/55)**	--------	3	0

## Discussion

The usage of antiseptic agents without following instructions, both in hospitals and the community, contributes to the spread of antiseptic resistance, which has become a major public health problem worldwide. This resistance may be due to plasmids carrying resistance genes such as *qacA/B, smr, qacG, qacH*, and *qacJ.* CoNS strains are exposed to selective pressure in the hospital setting, leading to their persistence and the potential to cause infections while also transferring their resistance genes to other pathogens, including *S. aureus* [9, 14, 22]. In this study, we examined the susceptibility of CoNS to various antiseptics commonly used in Egyptian hospitals, aiming to provide crucial information on the rate of spread of resistant strains.

The healthcare environment is increasingly recognized as a reservoir of multidrug-resistant (MDR) bacteria. From the environment, these bacteria can be transmitted by the hands of healthcare providers, colonize the skin and mucous membranes of patients, and subsequently cause healthcare-associated infections [23]. Therefore, we collected samples from the hospital environment, healthcare workers, and infected patients. Among the collected isolates, we identified 55 CoNS isolates and investigated their decreased susceptibility to three commonly used antiseptics: BC, CTAB, and CHDG, with resistance rates of 94.5%, 96.4%, and 80%, respectively. In Iran, CoNS clinical isolates showed a resistance rate to CHDG of 18.9%, with less susceptibility to BC [24]. Similarly, *Staphylococcus* *spp*. clinical samples (mostly CoNS) in a Turkish university hospital exhibited reduced susceptibility to BC (40.6%) and CHDG (33.3%) [14]. Additionally, 31% of CoNS clinical isolates from a Brazilian hospital displayed reduced susceptibility to chlorhexidine, which was attributed to the presence of antiseptic resistance genes [25]. This notable difference in resistance rates may be due to the widespread use of CHDG in Egyptian hospitals, leading to selective pressure and the persistence of CoNS in the hospital environment.

Epidemiological studies on antiseptic resistance genes have become essential due to their increased prevalence in various staphylococcal species [9]. In our study, the rate of *qacA/B* genes was 92.7% among all CoNS isolates and was detected in all CoNS clinical isolates. This was remarkably higher than the rates detected in CoNS clinical isolates from Iran and Sri Lanka, which were 47.1% and 16%, respectively [24, 26]. The increasing rates over the years are likely due to the excessive usage of various QACs and chlorhexidine in hospitals without following instructions, leading to an increase in antiseptic resistance genes on multi-resistance plasmids [1].

In our study, nearly all antiseptic resistance genes, such as *smr, qacG, qacJ*, and *qacH*, were more prevalent in isolates from colonized healthcare workers than in clinical and environmental isolates. The difference was significant regarding the *smr* and *qacJ* genes (P value ˂0.032 and P ˂0.001, respectively). This can be attributed to the excessive usage of various antiseptics by doctors and nurses in hospitals, sometimes without following instructions, which increases the prevalence of antiseptic resistance genes.

The overuse of QACs and biguanides may lead to an increase in drug resistance and a decrease in microbial diversity for some species, as different strains of the same species may have different adaptations to various antiseptics. As a result, strains with the highest adaptation will become more abundant than others [11]. Among samples from healthcare workers, especially from nurses, we found two isolates containing four antiseptic resistance genes (*qac A/B, smr, qac H, qacJ*), indicating the highest level of antiseptic resistance. In contrast, a study from Turkey found that CoNS clinical samples had no more than three genes in the same sample [14]. These isolates belonged to *S. epidermidis* and *S. haemolyticus*, both of which have a high ability to adapt to antimicrobial agents, enabling their survival and increased abundance compared to other species in the future.

Additionally, differences in the prevalence of antiseptic resistance genes between countries or within different hospitals in the same country can be attributed to variations in antimicrobial policies in each hospital and the types of CoNS species colonizing the community.

We attempted to find a possible association between reduced susceptibility to antiseptics and the presence of antiseptic resistance genes in CoNS isolates. Some variations were observed between phenotypic susceptibility (MIC results) and the detection of antiseptic resistance genes by PCR. Approximately 5.5% of CoNS isolates had no antiseptic resistance genes but showed reduced susceptibility to antiseptics (BC, CTAB, and CHDG). This finding was similar to another study from Saudi Arabia where 2% of chlorhexidine-resistant CoNS, as determined by MIC test, had no antiseptic resistance genes by PCR [1]. This reduced susceptibility may be attributed to the presence of other antiseptic resistance genes that encode other efflux pump proteins, such as NorA, NorB, LmrS, MdeA, and MepA [11], which were not investigated.

In our study, some CoNS isolates were sensitive to antiseptics despite containing at least one antiseptic resistance gene. A similar observation was reported for CoNS clinical isolates from Brazilian hospitals, which were positive for the *qacA/B* gene but sensitive to chlorhexidine [25]. The presence of genes does not guarantee gene expression, as expression is affected by transcription regulators, degree of exposure, and excessive contact with cationic agents without following instructions.

A high rate of MDR-CoNS isolates, defined as “non-susceptible to at least one agent in three or more antimicrobial categories,“ was observed in this study, consistent with the results of previous studies on CoNS isolates from clinical infections in Brazilian hospitals, where MDR was reported [25, 27]. In contrast, another study from Ethiopia indicated lower rates of antimicrobial resistance among CoNS isolates [28]. The higher rate of resistance in this study may be due to higher exposure, excessive use of antimicrobial agents, and deficiencies in infection control policies [29].

In the present study, all CoNS isolates were susceptible to linezolid (100%). This finding aligns with data from *S. epidermidis* clinical isolates from different wards of a children’s hospital in Tehran [30], suggesting that linezolid could be considered an effective antibiotic agent against CoNS.

MR-CoNS have been reported since the early 1980s, with increasing rates over the years [14]. In our study, CoNS isolates (based on the presence of the *mecA* gene) were divided into MS-CoNS (36.4%) and MR-CoNS (63.6%) isolates. In another study, the overall MR rate among CoNS clinical isolates from hospitals in Saudi Arabia was 32.1% using the same method [1]. Among the MS-CoNS in the current study, 85% contained one or more antiseptic resistance genes, while all MR-CoNS isolates exhibited at least one resistance gene. Ignak and colleagues reported that 67.5% of CoNS from clinical samples were MR-CoNS harbouring at least one antiseptic resistance gene [14]. This suggests that methicillin resistance increases the probability of the presence of antiseptic resistance genes.

Furthermore, in our study, all MS-CoNS lacked *mupA*, and only one isolate (5%) had *mupB*. In contrast, MR-CoNS had *mupA* in 20% of isolates and *mupB* in 25.7% of isolates. Among CoNS clinical isolates from Canada, 22.2% of MS-CoNS and 75% of MR-CoNS had *mupA*, and no *mupB* was detected [9]. This reflects that the mupirocin resistance rate is higher in MR-CoNS than in MS-CoNS.

Various approaches exist to examine the occurrence of methicillin resistance in both *S. aureus* and CoNS isolates. In this investigation, the cefoxitin disc diffusion technique was employed in accordance with CLSI guidelines [15], employing *S. aureus* ATCC 25923 as a reference control. The results indicated a prevalence of 65.6% (36/55) for MR-CoNS among the examined samples. Furthermore, the utilization of PCR analysis for *mecA* gene detection highlighted that 63.7% (35/55) of CoNS isolates were methicillin-resistant. These findings harmonize with prior research [31–33].

Other research has indicated that the disc diffusion test can yield false-negative results, especially for strains with heterogeneous resistance [34–37]. Our findings demonstrate that the disc diffusion method can also lead to false-positive results, consistent with prior studies [37, 38].

It is suggested that cefoxitin disc diffusion test-positive but PCR-negative isolates may be penicillinase hyperproducers, leading to the hydrolysis of penicillinase-resistant penicillins. These strains show a reduction or borderline susceptibility to oxacillin, referred to as “borderline oxacillin-resistant *S. aureus*”. The borderline phenotypes have been linked to alternative mechanisms, including inducible, plasmid-mediated methicillinase production, and alterations in penicillin-binding protein genes due to spontaneous amino acid substitutions in the transpeptidase domain [39–41]. Distinguishing these low-level resistant bacteria from true resistant strains containing the *mecA* gene can pose a challenge. Therefore, precise differentiation of MR-CoNS requires the detection of the *mecA* gene, and PCR can be a valuable method in clinical laboratories. Additionally, methicillin resistance in these isolates might be due to the presence of other genes, such as *mecB* or *mecC* [42].

We attempted to find a possible association between the presence of antiseptic resistance genes and mupirocin resistance genes in CoNS isolates. Our results showed that both *mup* and *qac* genes were present in 27.2% of CoNS (clinical, healthcare workers, and environmental) isolates that were positive for either *mupA* or *mupB* or both with *qac*’s and *smr* genes. This co-existence can be a critical risk factor for decolonization failure. These findings are in concordance with a previously published report in which the co-existence of *mupA* with *qacA/B* among CoNS clinical isolates from hospitals in Saudi Arabia was reported [1].

Twenty-five CoNS isolates, which were highly resistant to antiseptics and antibiotics, were selected for identification using the API-Staph system. *S. epidermidis* was the most frequently isolated species, followed by *S. chromogenes, S. haemolyticus*, and *S. hominis*. Among CoNS isolates, *S. epidermidis, S. haemolyticus*, and *S. hominis* are known to cause human infections [43]. *S. chromogenes* is not typically found in the human microflora; it is rare in humans but is considered the most common cause of bovine mastitis, an important disease affecting dairy animal productivity [44]. The detection of *S. chromogenes* in our hospital reflects the rural nature of our community, where villagers can carry *S. chromogenes* to hospitals.

In this study, we used water-based (aqueous) formulas rather than alcohol-based (tincture) formulas for all tested antiseptics to exclude the bactericidal effect of alcohol. The low number of clinical isolates compared to other types is a limitation in this study, which was due to the impact of the COVID-19 pandemic on the healthcare sector, where empiric management was adopted for most infection cases.

## Conclusion

Based on our work, we have concluded that antiseptic resistance genes were highly prevalent among CoNS isolated from healthcare workers, while antibiotic resistance was highly prevalent among clinical infection samples. CHDG exhibited higher activity compared to BC and CTAB in our hospital. Future studies should consider investigating the combined effect of these antiseptics. Further research on *S. chromogenes* in the hospital environment and the mechanism of its resistance to antiseptics and antibiotics is recommended. Additionally, more attention should be paid to storage, preparation, dilution, and use of antiseptics as instructed by the manufacturers.

### Electronic supplementary material

Below is the link to the electronic supplementary material.

Supplementary Material 1

## Data Availability

Data for this study may be made available upon request from the corresponding author.

## Abbreviations

AMC: Amoxicillin/clavulanic acid
AMP: Ampicillin
BC: Benzalkonium chloride
C: Chloramphenicol
CAS: Chemical Abstracts Service
CD: Clindamycin
CHDG: Chlorhexidine digluconate
CIP: Ciprofloxacin
CLSI: Clinical and Laboratory Standards Institute
CoNS: Coagulase Negative *Staphylococci*
COT: Trimethoprim/sulfamethoxazole
CTAB: Cetyltrimethylammonium bromide
CX: Cefoxitin
E: Erythromycin
EUCAST: European Committee on Antimicrobial Susceptibility Testing
GEN: Gentamicin
IPM: Imipenem
LZ: Linezolid
MDR: Multidrug-resistanance
MHB: Mueller–Hinton broth
MIC: Minimum inhibitory concentration
MR-CoNS: Methicillin-resistant - coagulase-negative staphylococci
MSA: Mannitol salt agar
MS-CoNS: Methicillin susceptible - coagulase-negative *staphylococci*
OX: Oxacillin
P: Penicillin G
PCR: Polymerase chain reaction
QACs: Quaternary ammonium compounds
RIF: Rifampicin
*S.*: *Staphylococcus*
TE: Tetracycline
TEI: Teicoplanin
