# Supplementary material for: Antiseptics and mupirocin resistance in clinical, environmental, and colonizing coagulase negative Staphylococcus isolates
Source: Antimicrob Resist Infect Control. 2023 Oct 4;12:110. doi: 10.1186/s13756-023-01310-3 (PMC10552417; doi:10.1186/s13756-023-01310-3)
Supplement: Supplementary file 1 — Supplementary Material 1 [file 13756_2023_1310_MOESM1_ESM.docx]

**Additional Table (1):** **Primers used for CoNS identification and** **methicillin resistance, mupirocin resistance and antiseptic resistance genes by PCR**

| Gene target | Primer | Sequence | Product size  (bp) | Ref |
| --- | --- | --- | --- | --- |
| *Staphylococcus*  *16S rRNA* | *Staph756F*  *Staph750R* | AACTCTGTTATTAGGGAAGAACA  CCACCTTCCTCCGGTTTGTCACC | 756 | **[9]** |
| *Nuc* | *nuc1*  *nuc2* | GCGATTGATGGTGATACGGTT  AGCCAAGCCTTGACGAACTAAAGC | 279 | **[9]** |
| *mecA* | *mecA*147-F  *mecA*112-R | GTGAAGATATACCAAGTGATT  ATCAGTATTTCACCTTGTCCG | 112 | **[9]** |
| *qacA, qacB* | *qacAB*-F2  *qacAB*-R2 | GCAGAAAGTGCAGAGTTCG  CCAGTCCAATCATGCCTG | 361 | **[9]** |
| *Smr* | *smr*-F2  *smr*-R2 | GCCATAAGTACTGAAGTTATTGGA  GACTACGGTTGTTAAGACTAAACCT | 195 | **[9]** |
| *mupA* | *mupA*  *mupB* | TATATTATGCGATGGAAGGTTGG  AATAAAATCAGCTGGAAAGTGTTG | 456 | **[9]** |
| *mupB* | *mupB*-F  *mupB*-R | CTAGAAGTCGATTTTGGAGTAG  AGTGTCTAAAATGATAAGACGATC | 674 | **[9]** |
| *qacG* | ***qacG*** **F**  ***qacG*** **R** | CAA CAG AAA TAA TCG GAA CT  TAC ATT TAA GAG CAC TAC A | 275 | **[14]** |
| *qacH* | ***qacH*** **F**  ***qacH*** **R** | ATA GTC AGT GAA GTA ATA G  AGT GTG ATG ATC CGA ATG T | 295 | **[14]** |
| *qacJ* | ***qacJ*** **F**  ***qacJ*** **R** | CTT ATA TTT AGT AAT AGC G  GAT CCA AAA ACG TTA AGA | 301 | **[14]** |

**Additional Table (2): Information about clinical isolates**

| **Serial N⁰** | **Sex** | **Department** | **Age**  **(years)** | **Specimen** | **Isolates** |
| --- | --- | --- | --- | --- | --- |
| 1 | male | Surgical ICU | 50 | BLOOD | CONS(MR) |
| 2 | female | Surgical ICU | 69 | BLOOD | CONS(MR) |
| 3 | male | Medical ICU | 24 | BLOOD | CONS(MR) |
| 4 | female | Medical ICU | 24 | BLOOD | CONS(MR) |
| 5 | female | Gynecology | 35 | Abscess | CONS(MR) |
| 6 | female | Internal Medicine | 19 | Abscess | CONS(MS) |
| 7 | male | Medical ICU | 70 | BLOOD | CONS(MR) |
| 8 | male | Surgical ICU | 72 | BLOOD | CONS(MR) |
| 9 | female | Orthopedic | 39 | Abscess | CONS(MR) |

CONS, Coagulase Negative *Staphylococcus*; MR, methicillin resistant; MS, methicillin sensitive

**Additional Table (3):** Antibiotic susceptibility for CoNS isolates

|  | **Sensitive** | **Intermittent** | **Resistant** |
| --- | --- | --- | --- |
| Antibiotics | N(%) | N(%) | N(%) |
| **Penicillin G(P) (10µg)** | 0 | 0 | 55(100.0) |
| **Cefoxitin (CX) (30µg)** | 19 (34.5) | 0 | 36 (65.5) |
| **Oxacillin (OX) (1µg)** | 32 (58.2) | 0 | 23 (41.8) |
| **Erythromycin(E) (15µg)** | 11 (20.0) | 12 (21.8) | 32 (58.2) |
| **Clindamycin (CD) (2µg)** | 38 (69.1) | 4 (7.3) | 13 (23.6) |
| **Trimethoprim/sulfameth oxazole(COT) (25µg)** | 42 (76.4) | 2 (3.6) | 11 (20.0) |
| **Ciprofloxacin (CIP) (5µg)** | 45 (81.8) | 2 (3.6) | 8 (14.5) |
| **Tetracycline (TE) (30µg)** | 47 (85.5) | 2 (3.6) | 6 (10.9) |
| **Rifampicin (RIF) (5µg)** | 47 (85.5) | 3 (5.5) | 5 (9.1) |
| **Gentamicin (GEN) (10µg)** | 51 (92.7) | 0 | 4 (7.3) |
| **Cloramaphenicol(C) (30µg)** | 53 (96.4) | 0 | 2 (3.6) |
| **Linezolid (LZ) (30µg)** | 55(100.0) | 0 | 0 |
| **Teicoplanin (TEI) (30µg)** | 52 (94.5) | 1 (1.8) | 2 (3.6) |
| **Imipenem (IPM) (10µg)** | 51 (92.7) | 0 | 4 (7.3) |
| **Ampicillin (AMP) (30µg)** | 3 (5.5) | 0 | 52 (94.5) |
| **Amoxicillin/Clavulanic acid (AMC) (20/10µg)** | 14 (25.5) | 1 (1.8) | 40 (72.7) |
| **Cefotaxime (CTX) (30µg)** | 18 (32.7) | 24 (43.6) | 13 (23.6) |

**Additional Table (4):** Antiseptic susceptibility between CONS isolates (N=55)

|  | **CONS isolates**  (N=55) | |
| --- | --- | --- |
|  | **Sensitive** | **Resistance** |
|  | N (%) | N (%) |
| **Benzalkonium chloride (BC)** | 3 (4.5) | 52 (94.5) |
| **Cetyltrimethy lammonium bromide (CTAB)** | 2 (3.6) | 53 (96.4) |
| **Chlorhexidine digluconate (CHDG)** | 11 (20) | 44 (80) |

CHDG resistance is defined as a MIC ≥ 4 µg/ml **[20];** CTAB resistance is defined as a MIC ≥0.5 µg/ml **[21]**, and BAC with a MIC> 3 µg/ml **[6]** was considered resistant.

**Additional Table (5):** Difference in antibiotic resistance between *S. epidermides* and non-epidermides CoNS.

|  | | ***S. epidermidis***  **(N=19)** | **Non-epidermidis CoNS.**  **(N=6)** | **P value** |
| --- | --- | --- | --- | --- |
| **Penicillin G (P)(10 µg) (N=25)** | N (%) | 19(100.0) | 6(100.0) | 0.0 |
| **Cefoxitin (CX)(30 µg) (N=21)** | N (%) | 11(58.0) | 5(83.3) | 0.65 |
| **Oxacillin (OX)(1 µg) (N=10)** | N (%) | 7(36.8) | 3(50.0) | 0.999 |
| **Erythromycin )E)(15 µg) (N=16)** | N (%) | 12(63.15) | 4(66.7) | 0.227 |
| **Clindamycin(CD)(2 µg) (N=10)** | N (%) | 6(18.8) | 4(28.6) | 0.003* |
| **Trimethoprim/sulfamethoxazole (COT)(25 µg) (N=7)** | N (%) | 2(10.5) | 5(35.7) | 0.125 |
| **Ciprofloxacin(CIP)(5 µg) (N=5)** | N (%) | 4(21.1) | 1(16.7) | 0.999 |
| **Tetracycline(TE)(30 µg) (N=3)** | N (%) | 3(15.7) | 0 | 0.54 |
| **Rifampicin(RIF)(5 µg) (N=3)** | N (%) | 3(15.8) | 0 | 0.55 |
| **Gentamicin(GEN)(10 µg) (N=4)** | N (%) | 3(15.8) | 1(16.7) | 0.24 |
| **Cloramaphenicol (C)(30 µg) (N=2)** | N (%) | 2(6.3) | 0 | 0.5 |
| **linezolid(LZ)(30 µg) (N=0)** | N (%) | 0 | 0 |  |
| **Teicoplanin (TEI)(30 µg) (N=1)** | N (%) | 0 | 1(16.7) |  |
| **Imipenem (IPM)(10 µg) (N=2)** | N (%) | 1(5.3) | 1(16.7) | 0.43 |
| **Ampicillin(AMP)(30 µg) (N=23)** | N (%) | 18(94.7) | 5(83.3) | 0.43 |
| **Amoxicillin/clavulanic acid (AMC) (20/10 µg) (N=19)** | N (%) | 15(78.9) | 4(78.9) | 0.6 |
| **Cefotaxime(CTX µg)(30) (N=17)** | N (%) | 13(68.4) | 4(66.7) | >0.99 |

*Significant
